# New specimens and molecular data provide validation of *Apatemon jamiesoni* n. sp. (Trematoda: Strigeidae) from water birds in New Zealand

**DOI:** 10.1007/s11230-022-10043-7

**Published:** 2022-05-24

**Authors:** Bronwen Presswell

**Affiliations:** Department of Zoology, University of Otago, PO Box 56, Dunedin, New Zealand

## Abstract

A study published in 2016 reported on an undescribed species of *Apatemon* (Strigeidae) from New Zealand that was previously well known from its larval stages. Only a single specimen from a mallard duck was available at the time, which was described and given the provisional name *Apatemon* sp. “jamiesoni”. Specimens also obtained from a spotted shag were not in good enough condition to form the basis of a new species description. A black-backed gull has since been discovered with specimens of this strigeid, their identity confirmed by genetic similarity, allowing formal description and naming of this species. This paper provides a description of the new specimens from the black-backed gull, along with a comparison with the specimens from other bird hosts, reprises some data from Blasco-Costa et al. (Parasitol Res 115:271–289, 2016) and presents formally the name *Apatemon jamiesoni* **n. sp.** This species differs from all other species of *Apatemon* in its small size, particularly that of the ovary and testes. It is most similar to *A. jamesi* from which it differs in the size of the oral and ventral suckers.

## Introduction

A study published in 2016 (Blasco-Costa et al. 2016) reported on an undescribed species of *Apatemon* Szidat, 1928 (Strigeidae) from New Zealand, that was nevertheless previously well-known from its larval stages (references listed under Synonyms). Unfortunately only a single specimen from a mallard was available at the time, which was described and figured, and given the provisional name *Apatemon* sp. “jamiesoni” until more specimens were found in order to establish the species. At the time specimens were also reported from a spotted shag, but these were not in sufficiently good condition to form the basis of a new species description. A third bird species, a black-backed gull, has since been discovered to harbour specimens of this strigeid, their identity confirmed by genetic similarity, and the author is now in a position to complete the description and formally name the species.

This species was originally familiar in New Zealand as a distinctive metacercarial cyst found in large numbers in common (*Gobiomorphus cotidianus* McDowall) and upland (*G. breviceps* (Stokell)) bullies (Actinopterygii: Eleotridae), and as cercariae from the freshwater snail, *Potamopyrgus antipodarum* (Gray) (Gastropoda: Tateidae). These hosts inhabit the fresh to brackish waters of coastal and inland lakes and streams of South Island, New Zealand. Cercariae have been reported from *P. antipodarum* in North Island (Hechinger 2012), although identity with the South Island species has not been confirmed genetically.

This paper provides a description of the new specimens from the black-backed gull, along with a comparison with the specimens from other bird hosts that match genetically, reprises some data from Blasco-Costa et al. (2016) and presents formally the name *Apatemon jamiesoni* **n. sp.** A phylogenetic tree is presented that confirms the identity of the specimens from the different hosts described herein, in the context of known strigeid sequences.

## Materials and methods

*Collection of material.* An adult mallard duck, *Anas platyrhynchos* L., was found as roadkill in the Mount Watkin region of Otago (2013), a spotted shag, *Phalacrocorax punctatus* (Sparmann), was found dead in Otago Harbour (2014) and a black-backed gull, *Larus dominicanus* Lichtenstein*,* was donated by the Dunedin Wildlife Hospital where it had died (2019). Viscera were dissected and intestinal worms were preserved in either 96 % ethanol for molecular analyses or 70 % ethanol for morphological study.

*Morphological analysis* Trematodes were stained using iron acetocarmine, dehydrated through a graded ethanol series, cleared in clove oil and mounted in Canada balsam. Measurements were made using ImageJ software (Wayne Rasband, NIH, USA) from photographs taken on an Olympus BX51 compound microscope mounted with DP25 camera attachment. Drawings were made with the aid of a drawing tube mounted on an Olympus compound microscope. Note that in the descriptions and tables the terms “prosoma” and “opisthoma” are used to replace the formerly used “forebody” and “hindbody”, following Achatz et al. (2021).

*Molecular analysis. *New DNA sequence of this *Apatemon* species was obtained from a black-backed gull and two common bullies. Sequences were also generated from two specimens of *Diplostomum*, one from the eye of a common bully (“humour metacercaria” of Ruehle et al. 2021) and one from a Caspian tern from Otago (*D. spathaceum* (Rudolphi, 1819)), which were collected during a different study, and used here as additional diplostomid data. Genomic DNA was extracted using the Dneasy Blood & Tissue Kit (Qiagen, Hilden, Germany) according to the manufacturer’s protocol. The D2 domain of the large subunit ribosomal DNA (28S) was amplified using primers T16 (5′ GAGACCGATAGC GAAACAAGTAC 3′) and T30 (5′ TGTTA GACTCCTT GGTCCGTG 3′) (Harper & Saunders, 2001) and conditions of 94°C for 5 min, 38 cycles of 94°C for 30 sec, 45°C for 30 sec and 72°C for 2 min, and 72°C for 7 min. All polymerase chain reaction (PCR) products were cleaned using EXOSAP-TM™ Express PCR Product Cleanup Reagent (USB Corporation, Cleveland, OH, USA), following manufacturer’s instructions. Sanger sequencing by capillary electrophoresis was performed by the Genetic Analysis Service, Department of Anatomy, University of Otago (Dunedin, New Zealand). Sequences were imported into Geneious Prime® version 1.2, trimmed using the trim function with default parameters and manually edited for incorrect or ambiguous base calls. They were aligned, together with published sequences of strigeids from GenBank (Table 1), using MAFT alignment tool in Geneious Prime. Bayesian inference analysis was conducted with MrBayes version 3.2.6 using the online interface Cyberinfrastructure for Phylogenetic Research (CIPRES) Science Gateway (Miller et al. 2010). Each analysis used random starting trees for 2 runs (each 1 cold and 3 heated chains), employing a Markov chain Monte Carlo (MCMC) approach for sampling the posterior probability distribution across 10,000,000 generations, sampling every 1,000 generations. The first and last 25% of samples were discarded as “burn-in”. Mixing and convergence of each run were monitored through statistics provided in Mr. Bayes and Tracer v1.6.0 (Rambaut et al., 2014). The resulting phylogenies were summarized in a 50% majority-rule consensus tree with clade credibility support values (Bayesian posterior probability, bpp) and branch length information; bbp higher than 0.95 was considered strong support for nodal positions. Genetic divergence was estimated in Geneious. Newly generated sequences were submitted to GenBank under accessions OM949805-9.

**Table 1:** Sequences (28S) used in phylogenetic analysis. GenBank numbers in **bold** were produced for this study

	Host	Locality	GenBank no.
*Alaria alata*	Racoon dog *Nyctereutes procyonoides*	Ukraine	AF184263
*Apatemon* cf. *hypseleotris*	Gudgeon *Hypseleotris* sp.	Australia	MT603884
*Apatemon gracilis*	Stickleback *Gasterosteus aculeatus*	Norway	KY513177
*Apatemon jamiesoni* **n. sp.**	Black backed gull *Larus dominicanus*	NZ	**OM949809**
	Bully A *Gobiomorphus cotidianus*	NZ	**OM949808**
	Bully B *Gobiomorphus cotidianus*	NZ	**OM949807**
	Mallard *Anas platyrhynchos*	NZ	KT334168
	Snail *Potamopyrgus antipodarum*	NZ	KT334166
	Spotted shag *Phalacrocorax punctatus*	NZ	KT334169
*Apatemon* sp.	NZ scaup *Aythya novaeseelandiae*	NZ	unpublished
*Apatemon* sp.	Snail *Radix auricularia*	Japan	LC599501
*Apatemon* sp.	Stickleback *Gasterosteus aculeatus*	Norway	KY513179
*Apharyngostrigea cornu*	Night heron *Nycticorax nycticorax*	Mexico	MF398345
*Apharyngostrigea pipentis*	Frog *Lithobates sylvaticus*	USA	JF820598
*Australapatemon burti*	Mexican duck *Anas diazi*	Canada	MF398342
*Australapatemon sp.*	Pintail duck *Anas acuta*	Canada	MF124270
*Australapatemon* sp.	Snail *Planorbella* sp.	USA	OK184706
*Australapatemon* sp. 1	Leech *Haemopis sanguisuga*	Poland	MW244634
*Australapatemon* sp. 2	Gadwall *Mareca strepera*	Poland	MW244635
*Australaptemon niewiadomski*	Mallard *Anas platyrhynchos*	NZ	KT334165
*Australpatemon* sp.	Snail *Planorbis planorbis*	France	MK168735
*Cardiocephaloides longicollis*	Gull *Chroicocephalus ridibundus*	Ukraine	AY222171
*Cardiocephaloides ovicorpus*	Little pied shag *Microcarbo melanoceucos*	NZ	MW481314
*Diplostomum phoxini*	Minnow *Phoxinus phoxinus*	Wales	AY222173
*Diplostomum* sp.	Bully *Gobiomorphus cotidianus*	NZ	**OM949806**
*Diplostomum spathaceum*	Caspian tern *Hydroprogne caspia*	NZ	**OM949805**
*Ichthyocotylurus erraticus*	Whitefish *Coregonus autumnalis*	N. Ireland	AY222172
*Nematostrigea serpens*	Osprey *Pandion haliaetus*	Russia	KF434762
*Parastrigea brasiliana*	Heron *Cochlearius cochlearius*	Mexico	MZ614713

## Results

**Fig. 1:**
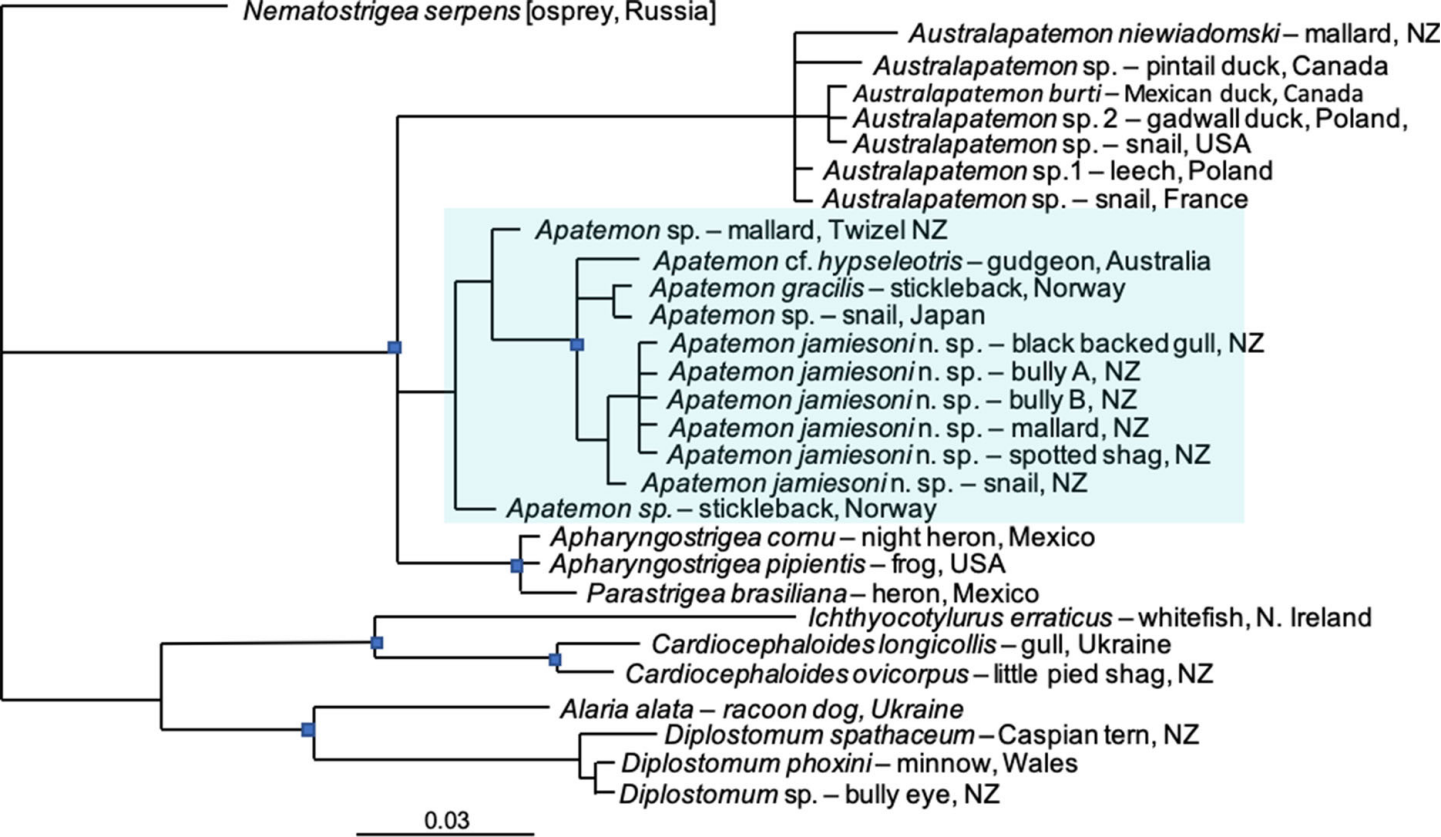
Bayesian 50% majority rule phylogenetic inference of the 28S data set. *Apatemon* sequences are included in the shaded box. Scale bar indicates the number of substitutions per site

In the phylogeny (Fig.1) based on 28S data (563bp) *A. jamiesoni* **n. sp.** sequences from the black-backed gull, bullies, mallard, spotted shag and *P. antipodarum* formed a monophyletic clade. *A. jamiesoni* **n. sp.** formed a trichotomy with *Apatemon* cf. *hypseleotris* from Australia, and *A. gracilis* from Norway and an unnamed species of *Apatemon* from Japan, support for which was strong. Sister to these were an unknown species from a mallard duck in New Zealand, and another from Norway. This clade combined with one containing *Australapatemon* spp., and one with *Apharyngostrigea* spp., to form an unresolved trichotomy. This phylogeny further supports the standing of *Apatemon* and *Australapatemon* as distinct genera (Blasco-Costa et al. 2016). *Parastrigea brasiliana* which sits uncomfortably within the *Apharyngostrigea* clade, has recently been moved to *Apharyngostrigea* (López-Jiménez et al. 2022), but we have retained the original GenBank name in the tree for ease of reference.

A single A/G dimorphism produced internal structure in the *Apatemon* clade, in which the sequences were otherwise identical. Interspecific divergence between *Apatemon jamiesoni* **n. sp.** and other *Apatemon* species ranged from 0.4% to 1.6%. Average genetic divergence between species of *Apatemon* and *Australapatemon* was 4.9% for the 28S marker, which fell within the range of variation of other strigeid genera (1.7-9.0%).

Unfortunately, attempts to sequence internal transcribed spacers (ITS1&2) and cytochrome oxidase 1 (*cox1*) markers were unsuccessful, so it is not possible to present phylogenies for these markers at this time. However, the new 28S phylogeny presented here includes additional samples to that of Blasco-Costa et al. (2016) and demonstrates the genetic identity of the new specimens and their relationship to other genetically characterised species.

**Family Strigeidae Railliet, 1919**

**Subfamily Strigeinae Railliet, 1919**

**Genus** ***Apatemon*** **Szidat, 1928**

***Apatemon jamiesoni*** **n. sp.**

Synonyms: *Apatemon* sp. in Blasco-Costa et al. (2013, 2015), Hammond-Tooke et al. (2012), Hechinger (2012), Herrmann and Poulin (2011a, b, 2012a, b), Hock and Poulin (2012), Kelly et al. (2009), Lagrue and Poulin (2015), Poulin (2013), Ruehle & Poulin (2019); Cercaria F1 of Winterbourn (1974); *Apatemon* sp. “jamiesoni” in Blasco-Costa et al. (2016), Blasco-Costa & Locke (2017), Presswell & Blasco-Costa (2020), Presswell and Bennett (2022a, b).

*Type host.* Black-backed gull, *Larus dominicanus* Lichtenstein

*Other definitive hosts*. *Anas platyrhynchos* L. and *Phalacrocorax punctatus* (Sparrman)

*Type locality.* Long Beach, Otago, New Zealand (45°75’54” S, 170°64’25” E).

*Other localities.* Mount Watkin, Otago (45°33′ S, 170°34′ E) (mallard); Otago Harbour (45°79′ S, 170°71′ E) (spotted shag); Tomahawk Lagoon, Otago (45°54′ S, 170°32′ E) (snail and bully); Lake Waihola and Waipori, Otago (46°01′ S, 170°05′ E and 45°58′ S, 170°06′ E) (snail and bully); plus many other localities throughout New Zealand’s North and South Islands.

*Site of infection.* Intestine (definitive hosts); body cavity, mesenteries, muscles, liver, gonads, cranial cavity (second intermediate hosts); gonads (first intermediate host).

*Second intermediate host*. *Gobiomorphus cotidianus* McDowall, *Gobiomorphus breviceps* (Stokell) and *Galaxias anomalus* (Stokell).

*First intermediate host. Potamopyrgus antipodarum* (Gray)

*Specimens collected.* Thirty-two from black-backed gull, 40 from spotted shag, 1 from mallard.

*Prevalence.* In one black-backed gull out of 50; in one spotted shag out of 35; in one out of 16 mallard; 100 % of 77 common bully from Lake Waihola; in 2 out of 3232 *P. antipodarum* from Lake Waihola (0.06 %).

*Intensity* in second intermediate host: 9–800 individuals, mean intensity 204, in common bully from LakeWaihola.

*Type material.* Holotype (slide) W.003617, Paratypes W.003618 (single slide) deposited with the Museum of New Zealand, Te Papa Tongarewa, Wellington, NZ.

*Voucher material*. Adult, Natural History Museum of Geneva, Switzerland, MHNG-PLAT-91644.

*Representative DNA sequences*: 28S rDNA, KT334166-KT334169 and OM949805-9; ITS1-5.8S-ITS2, KT334170-KT334172; COI, KT334181-KT334182.

*Zoobank reference:* urn:lsid:zoobank.org:pub:FCA4C38F-E733-49FE-A0E5-F2BEDED9A4D0

*Etymology.* The species is named in memory of the late Professor Ian Jamieson, friend and colleague, who through his research contributed enormously to bird conservation in New Zealand.

### Description (Fig. 2)

**Fig. 2:**
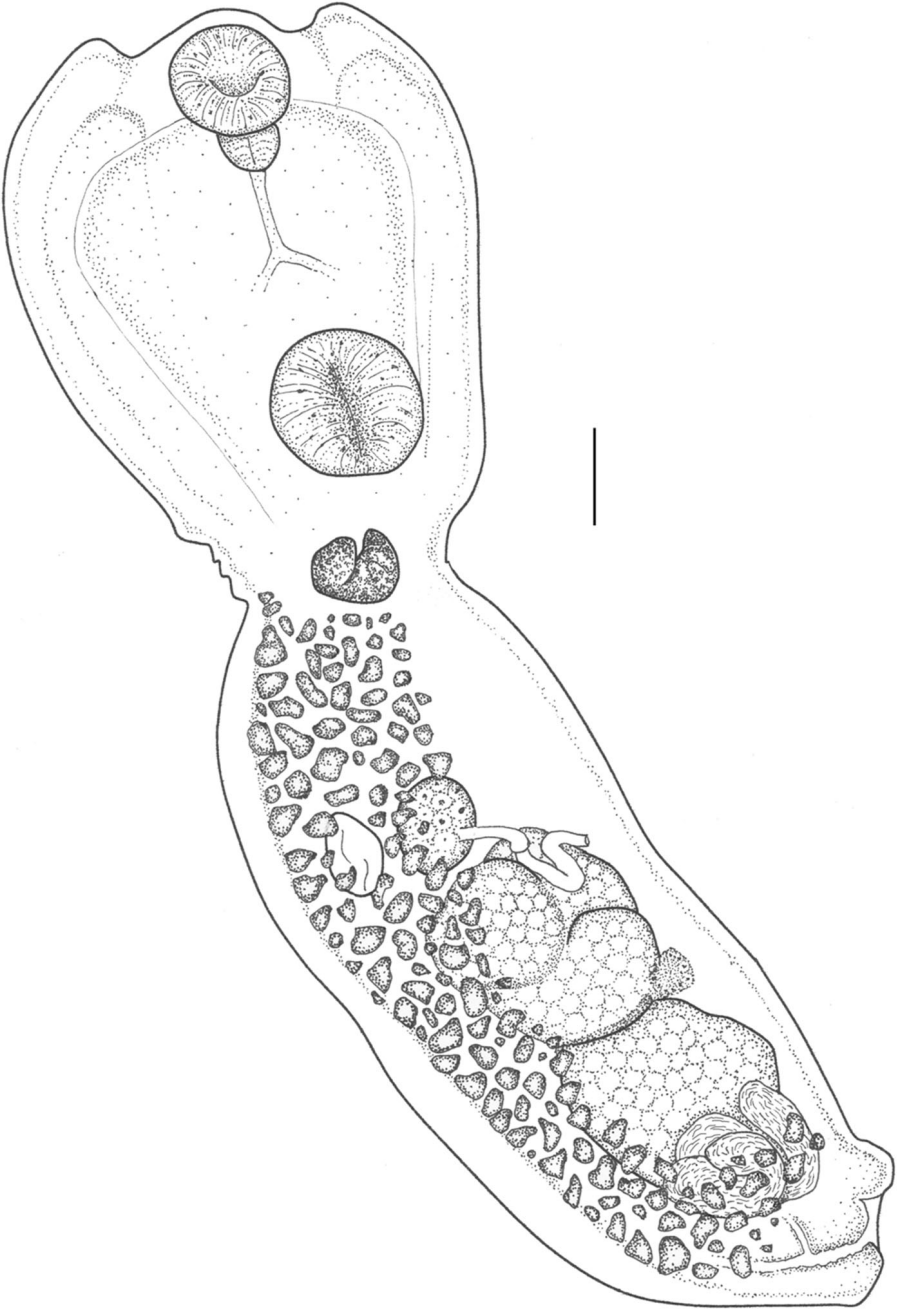
*Apatemon jamiesoni* **n. sp.** Holotype adult from black-backed gull *Larus dominicanus*. Scale bar = 100µm

[Based on holotype specimen from *Larus dominicanus* Lichtenstein. Measurements of all other specimens given in Table 2. Measurements in micrometres].

**Table 2:** Comparative metrical data for specimens of *Apatemon jamiesoni* **n. sp.** ex spotted shag *Phalacrocorax punctatus* (15 specimens), mallard *Anas platyrhynchos* (1 specimen)and black-backed gull *Larus dominicanus* (8 specimens)

	spotted shag			mallard	black-backed gull		
	Range	av	n	subadult	Range	av	n
Total length	1131–1537	1239.08	12	1348	1054–1776	1528.3	8
Prosoma length	394–522	445.77	14	545	445–714	612.5	8
Prosoma width	299–420	366.00	14	333	291–478	405.0	8
Opisthoma length	704–1133	782.58	12	803	675–1093	954.0	7
Opisthoma width	293–401	342.88	14	333	292–397	340.0	7
Oral sucker length	121–139	134.00	2	88	104–139	124.8	8
Oral sucker width	118–125	121.33	2	91	103–133	119.3	8
Pharynx length	51–72	60.00	3	58	49–84	69.6	8
Pharynx width	50–52	50.67	3	48	42–77	60.5	8
Vent sucker length	152–162	157.33	6	148	148–189	158.6	8
Vent sucker width	153–169	162.25	4	139	140–180	157.8	8
Prot Glands length	46–76	57.30	10	45	47–78	62.6	6
Prot Glands width	78–105	94.20	10	94	83–106	92.0	6
Ovary length	71–96	83.86	7	82	61-85	74.3	7
Ovary width	110–134	124.57	7	130	95–133	109.7	7
Anterior testis length	157–190	174.00	4	167	132–221	185.8	7
Anterior testis width	160–208	183.25	4	152	141–212	175.3	7
Posterior testis length	154–201	175.00	4	176	132–234	179.5	7
Posterior testis width	150–195	164.25	4	136	125–241	185.9	7
Egg length	84.67–97.50	89.46	9	80	69–101	87.5	5
Egg width	46.67–57.00	51.74	9	49	49-62	54.7	5
Ratio prosoma to opisthoma	1:1.46–2.00	1:1.67	11	1:1.47	1:1.31–1.70	1:1.51	7
Ovary position %	25.10–32.85	27.84	5	23	15.41–26.53	21.86	5
Anterior Testis position %	36.48–43.42	39.22	3	38	27.27–40.32	31.86	6
Posterior Testis position %	57.61–69.97	64.09	3	74	52.27–61.17	55.60	6
Oral:ventral sucker width ratio	1:1.30–1.36	1:1.33	3	1:1.53	1:1.24–1.48	1:1.33	8

Data from a single bird host in each case. n = number of worms from which data were taken

Total length 1677; body distinctly bipartite; maximum width between oral and ventral sucker. Tegument smooth. Prosoma cup-shaped; 663 long, 433 wide. Opisthoma subcylindrical; usually widest at level of anterior testis; 1022 long, 380 wide. Prosoma to opisthoma length ratio 1:1.54. Oral sucker subterminal; 132 long, 133 wide. Prepharynx absent. Pharynx 79 long, 63 wide. Ventral sucker in mid-prosoma; 170 long, 166 wide. Oral to ventral sucker length ratio 1: 1.25. Holdfast with two lobes; associated proteolytic gland at base of prosoma, level with, or slightly anterior to constriction; 78 long, 90 wide. Ovary smooth, transversely or slightly oblique elongate oval, median; 82 long, 105 wide. Ovary positioned 26.2 % length of opisthoma. Laurer’s canal long, broad and convoluted. Mehlis’ gland intertesticular. Testes tandem, large; anterior testis round to bi- or tri-lobed; anterior margin positioned 40.3% length of opisthoma; 219 long, 184 wide. Posterior testis asymmetrical, lobed; anterior margin positioned 61.2 % length of opisthoma; 210 long, 241 wide. Seminal vesicle highly convoluted, post-testicular. Vitellarium densely follicular, restricted to opisthoma, occupying preovarian region, extending posteriorly in a ventral field to level of copulatory bursa. Vitelline reservoir intertesticular; median. Uterus extends anterior to ovary, ventral to gonads, with up to 5 large eggs (holotype contains only one, collapsed, egg). Copulatory bursa small. Genital cone not delimited from surrounding parenchyma. Hermaphroditic duct only slightly rugose, opens close to apex of genital cone.

*Metacercaria and cercaria*

Full descriptions, illustrations and measurements of metacercariae from common bully *Gobiomorphus cotidianus* McDowall and cercariae from *Potamopyrgus antipodarum* (Gray) are given in Blasco-Costa et al. (2016).

### Remarks

In having a bipartite body, cup-shaped prosoma, sub-cylindrical opisthoma, vitellarium confined to opisthoma, tandem testes, a small genital cone poorly delimited from parenchyma, and ringnapf absent (Niewiadomska 2002), these specimens conformed to the genus *Apatemon*. A full comparison with other species of *Apatemon* is given in Blasco-Costa et al. (2016), including a table of measurements. The new specimens have increased the range of metrics for this species description (see Table 2), but the new ranges have little altered the comparative diagnosis when all eight valid *Apatemon* species and subspecies are considered.

The new specimens were compared against all congeners by consulting the original descriptions. *Apatemon jamiesoni* **n. sp.** is smaller in body size than *Apatemon gracilis* (Rudolphi, 1819) (as re-described by Dubois (1968)), *A. hypseleotris* Negm-Eldin & Davies, 2001, *A. somateriae somateriae* (Dubois, 1948), *A. s. fischeri* Dubois, 1968*, A. vitelliresiduus* Dubois & Angel, 1972, *A. buteonis* (Yamaguti, 1933) and *A. fuligulae* Yamaguti, 1933. Notably, the length of the ovary and size of the testes of *Apatemon jamiesoni* **n. sp**. lie below the range of all the above species. Likewise, *Apatemon jamiesoni* **n. sp.** measurements lie outside of those for the suckers and pharynx of *A. s. fischeri*. In addition, *A. vitelliresiduus* has vitellaria that extend into the prosoma and *A. buteonis* has post-equatorial testes and a ringnapf. Moreover, *A. gracilis* has metacercarial cysts that are lemon shaped as opposed to the egg-shaped cysts of *Apatemon jamiesoni* **n. sp.** and *A. hypseleotris* and *A. fuligulae* have notably smaller cysts.

The species that most closely resembles *Apatemon jamiesoni* **n. sp.** is *A. jamesi* Palmieri,

Krishnasamy & Sullivan, 1979. However, *A. jamesi* has smaller oral suckers (range 78–87 x 78–97) and ventral suckers (range 74-147 x 92-129), than *A. jamiesoni* **n. sp.** (88-139 x 99-133 and 148–189 x 139–180 respectively). Furthermore, *A. jamesi* is unusual in having a leech second intermediate host.

## Discussion

Blasco-Costa et al. (2016) took the prudent step of not publishing a formal naming of the *Apatemon* specimens found during their study due to a lack of suitably well conserved specimens. However, a full description of their single specimen was provided, along with descriptions and illustrations of the larval stages, and the provisional name *Apatemon* sp. “jamiesoni” was given, pending discovery of further specimens. In finding new specimens, defined by genetic identity with the original specimens from the mallard and the spotted shags, the author has hereby been able to complete the erection of the new species as *Apatemon jamiesoni* **n. sp.**

The new specimens described here raise a number of questions. Generally thought of as marine birds, the spotted shag and black-backed gull seem unlikely hosts of a parasite whose first and second intermediate hosts are found in fresh to brackish water. However, spotted shags are known to forage and roost in estuaries, and black-backed gulls in New Zealand are found anywhere from coastal waters to high-country farms, particularly during the breeding season (Szabo 2013; Miskelly 2013). An interesting observation is worthy of note here: *Apatemon jamiesoni* **n. sp.** specimens were found in only a single black-backed gull, which was also infected with *Cryptocotyle micromorpha* Presswell & Bennett, 2022a, 2022b, a gastrointestinal trematode only known from freshwater habitats. This was the only bird out of fifty that the author has examined to have parasites of a freshwater origin, underlining that, despite their cosmopolitan occurrence, black-backed gulls remain preferentially foragers of the sea shore. A further puzzle that occurs is the apparent rarity of *Apatemon jamiesoni* **n. sp.** in birds frequenting freshwater and brackish habitats. We have examined a large number of water fowl individuals from localities where the metacercariae are found in huge numbers in common bullies, and yet only in these three disparate birds have they been discovered as adults. The mallard was found in a terrestrial environment and the shag and gull are preferentially seabirds. A recent investigation into three specimens of Australasian crested grebe and one white-faced heron from Lake Wanaka (Presswell & Bennett 2022b) found thousands of metacercarial cysts and freshly excysted juveniles in the stomachs of both species, but none in the intestine, and no adults at all. Although DNA sequencing was not possible from those specimens it was proposed that they were the metacercariae of *Apatemon jamiesoni* **n. sp.** which were unable to mature in these two hosts. Which leaves the question, through which definitive host is this species of strigeid completing its lifecycle in large numbers, considering the high prevalence and intensity in bullies? Further collection of freshwater birds is needed to answer this question.

Finally, this new species, confirmed and named in this paper, represents only one more strigeid in the trematode list for New Zealand. Undoubtedly, as stated in Blasco-Costa et al. (2016) there are more discoveries to be made. The author’s collection includes a genetically distinct *Apatemon* sp. from another mallard found in Mackenzie District (included in phylogeny, Fig. 1) and a further morphological variant from New Zealand scaup, along with a species of *Strigea* from Australasian harriers, all of which have yet to be described.

## Data Availability

Not applicable as no datasets were generated or analysed during the study.
